# Controlled Catheter Movement Affects Dye Dispersal Volume in Agarose Gel Brain Phantoms

**DOI:** 10.3390/pharmaceutics12080753

**Published:** 2020-08-11

**Authors:** Jason N. Mehta, Gabrielle R. McRoberts, Christopher G. Rylander

**Affiliations:** 1 Walker Department of Mechanical Engineering, The University of Texas at Austin, 204 E. Dean Keeton Street, Stop C2200, Austin, TX 78712-1591, USA; jnmehta@utexas.edu; 2 Department of Neuroscience, The University of Texas at Austin, Austin, TX 78712-1591, USA; gabbymcroberts@gmail.com

**Keywords:** convection-enhanced delivery, dispersal volume, glioblastoma, catheter movement

## Abstract

The standard of care for treatment of glioblastoma results in a mean survival of only 12 to 15 months. Convection-enhanced delivery (CED) is an investigational therapy to treat glioblastoma that utilizes locoregional drug delivery via a small-caliber catheter placed into the brain parenchyma. Clinical trials have failed to reach their endpoints due to an inability of standard catheters to fully saturate the entire brain tumor and its margins. In this study, we examine the effects of controlled catheter movement on dye dispersal volume in agarose gel brain tissue phantoms. Four different catheter movement control protocols (stationary, continuous retraction, continuous insertion, and intermittent insertion) were applied for a single-port stepped catheter capable of intrainfusion movement. Infusions of indigo carmine dye into agarose gel brain tissue phantoms were conducted during the controlled catheter movement. The dispersal volume (*V*_d_), forward dispersal volume (*V*_df_), infusion radius, backflow distance, and forward flow distance were quantified for each catheter movement protocol using optical images recorded throughout the experiment. *V*_d_ and *V*_df_ for the retraction and intermittent insertion groups were significantly higher than the stationary group. The stationary group had a small but significantly larger infusion radius than either the retracting or the intermittent insertion groups. The stationary group had a greater backflow distance and lower forward flow distance than either the retraction or the intermittent insertion groups. Continuous retraction of catheters during CED treatments can result in larger *V*_d_ than traditional stationary catheters, which may be useful for improving the outcomes of CED treatment of glioblastoma. However, catheter design will be crucial in preventing backflow of infusate up the needle tract, which could significantly alter both the *V*_d_ and shape of the infusion.

## 1. Introduction

Glioblastoma is a highly infiltrative brain tumor with margins that typically extend centimeters beyond the visible tumor [1]. Even with treatment, patient survival is poor, with a five-year survival rate of 5.5% [2] and a median survival of 12 to 15 months [3]. Gold-standard treatment protocols involving surgical resection, radiotherapy, and concomitant or adjuvant chemotherapy are not effective and recurrence is unavoidable [4,5,6]. Even combinatory therapy, which uses multiple standard-of-care treatments, has resulted in only moderate improvement in overall patient survival [7], and the tumor generally recurs at or near the site of the original tumor location [8,9].

The presence of the blood–brain barrier (BBB) and blood–brain–tumor barrier (BBTB) make systemic delivery of therapeutics to the brain challenging. In order to bypass the BBB and overcome the limitations of diffusive delivery of drugs, Bobo et al. proposed the use of convection-enhanced delivery (CED) [10]. CED uses locoregional delivery of therapeutics to bypass the BBB by inserting a small caliber catheter though a burr hole in the skull and directly into the brain parenchyma. This technique utilizes pressure-driven flow to deliver large amounts of therapeutics directly to the region of interest [10,11]. CED infusions occur primarily within the interstitial space of the brain. Finally, drug infusion times can range from a few hours to several days [12].

Although treatment of malignant gliomas using CED appeared promising after phase I [13,14,15] and phase II [14] clinical trials, a phase III (PRECISE) trial failed to meet its clinical endpoints [16]. A retrospective study conducted by Sampson et al. examined possible reasons for the failures of the PRECISE (Phase III Randomized Evaluation of CED of IL13-PE3QQR with Survival Endpoint) trials and concluded that inadequate drug delivery volume to the tumor may have been an important indicator for the failure of the trial, as on average only 20.1% of the 2 cm penumbra surrounding the tumor resection cavity was covered by the drug [17]. Additionally, initial trials of CED treatment for Parkinson’s disease using a glial cell-line-derived neurotrophic factor (GDNF) showed promising results [18,19]. However, larger clinical trials failed to reach their primary endpoints [20], and a retrospective study found that only 2–9% of the target area was covered [21].

Standard catheters are unable to fully penetrate target volumes with CED treatments and have additionally been shown to be susceptible to reflux up the needle track, ultimately reducing the overall infusion volume [22,23]. In order to combat the low delivery volumes found in clinical trials, focus was directed to improving the catheter design. Many of these devices focused on limiting backflow using a step change [24,25,26,27,28] and adding multiple ports within a single primary cannula to increase total infusion volume [29,30]. However, exploration of alternative methods to increase the volume dispersed (*V*_d_), such as catheter movement during infusion, has been sparse. Breeze et al. initially proposed the use of retraction in order to achieve an elongated deposition while implanting fetal tissue in the putamen of patients with Parkinson’s disease [31]. No other groups have attempted to recreate the elongated geometry with continuous retraction but some clinical trials have relied on depositing multiple payloads of therapeutics to target the putamen to treat Parkinson’s disease [32,33,34]. Additionally, Sillay et al. examined two-step advancement and retraction of catheters in agarose phantoms [35]. The study indicated that advancement of the catheter resulted in less backflow and more spherical delivery than retraction. However, their results were dependent on the catheter geometry and they relied on manual catheter placement. Further, the primary measures were the radius of infusion and backflow of infusate rather than total *V*_d_. Bankiewicz et al. used single-step advancing catheters after an initial deposit in order to elicit backflow to reach over 50% of the putamen with a single catheter [36]. Further, Sudhakar et al. used an “infuse-as-you-go” technique, where real-time MRI was used to guide catheter advancements of between 2 and 4 mm in order to cover the putamen of non-human primates. “Infuse-as-you-go” covered nearly 15% more of the putamen in about 38% of the time compared to bilateral infusions via transfrontal trajectories [37]. Finally, Lewis et al. found that an increase in step length of the recessed step catheter would result in controlled reflux and an increase in *V*_d_ [38]. Unlike the previous work mentioned, in this study we examine the effects of continuous remote retraction and insertion, intermittent insertion, and stationary catheter infusions on *V*_d_ in agarose gel brain tissue phantoms.

## 2. Materials and Methods

Two 8-cm-long catheters were manufactured using 250 µm ID/375 µm OD fused silica capillary tubing (TSP250350, General Separation Technologies, Inc., Wilmington, DE, USA). The capillary tubing was cleaved flat and attached to a 22 gauge plastic dispensing tip using cyanoacrylate glue and UV-cured acrylic. Further, using a similar process to work previously conducted [39,40], a 50 µm solid core multimode optical fiber (M14L02, Thorlabs Inc., Newton, NJ, USA) was adhered to the annular wall of the capillary tube using cyanoacrylate glue, such that the two parts co-terminated. The addition of the multimode fiber made the catheters capable of both fluid and light energy delivery; however, the light delivery functionality was not examined in the present work.

Agarose gel has previously been shown to be a good model for healthy porcine brain tissue [41]; therefore, it was used as a brain tissue surrogate in this study. Agarose gel brain phantoms were prepared by creating an agarose solution (0.6% *w*/*w*) by mixing certified molecular biology agarose powder (Bio-Rad Laboratories, Hercules, CA, USA) and deionized water. The solution was heated to a low boil and continuously stirred until the mixture became optically clear. After a cooling period of 15 min, approximately 50 mL of agarose was poured into polystyrene molds, covered, and allowed to completely cool to room temperature (25 ± 2 °C).

**Figure 1:**
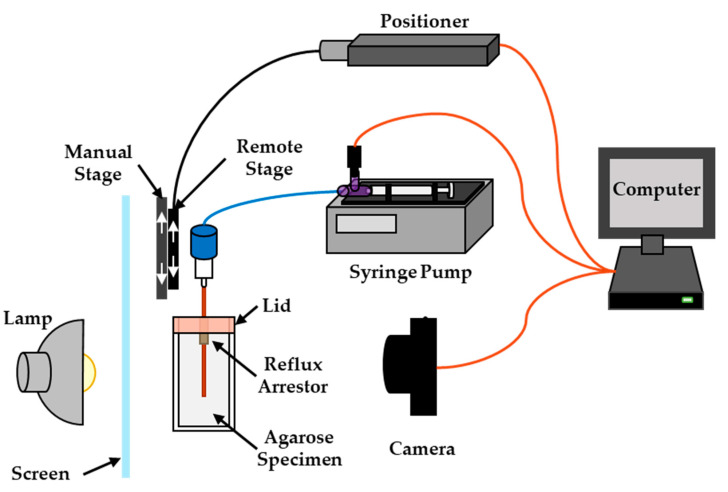
Schematic of shadowgraphy setup, where a linear positioner controls a stage used to move the catheter through a reflux arresting cannula into an agarose specimen. The manual stage allows for gross alignment of the catheter prior to insertion into the gel. A constant-flow syringe pump is used to deliver indigo carmine dye through the catheter and into the gel, while a camera is used to acquire images throughout the infusion.

Immediately prior to infusions, a custom acrylic lid with an integrated reflux arresting tube was placed on top of the agarose mold, such that the reflux arresting tip was inserted into the agarose, as shown in Figure 1. The reflux arresting tip was constructed from polyether-ether ketone (PEEK) tubing (1.01 mm ID/1.57 mm OD). This design allowed for the catheters to move relative to both the agarose and the reflux arresting tip, with both components remaining as fixed reference points.

A shadowgraphy setup similar to that used previously [29] allowed visualization of the dye *V*_d_. Briefly, two agarose gel phantoms were placed on an acrylic stage in front of a light-diffusing shade and behind a complementary metal-oxide-semiconductor camera (Rebel T1i, Cannon Inc., Ohta-ku, Tokyo, Japan), allowing for two experiments to be conducted at the same time. The agarose gel phantoms were kept at 25 °C, consistent with previous studies [29,38,41] and below the gel’s gelling temperature of 36 °C. Two catheters were supported by two linear stages in series—the first stage allowed for manual deployment, while the second stage allowed for remote deployment, as shown in Figure 1. For all groups, the catheters were first manually inserted into the gel phantoms through the reflux arresting tubing using the manual linear stage, such that they just began protruding from the reflux arresting tip. Quick insertion rates were shown to minimize reflux in agarose gel phantoms [42]; therefore, once the catheters were safely guided into the phantoms, they were rapidly deployed at 600 mm/min to their starting point. The catheters were controlled with a high-precision linear actuator (A-LAR-E series, Zaber Technologies, Vancouver, BC, Canada) via a Bowden cable attached to the linear stage containing the catheters. The actuator was controlled with a custom graphical user interface created using LabView (National Instruments, Austin, TX, USA). Four experimental groups were examined: (1) a standard stationary catheter acting as the control, (2) continuously retracting catheter, (3) continuously advancing catheter, and (4) intermittently advancing catheter. The stationary and continuously retracting catheters were inserted to a depth of 30 mm, whereas the continuously and intermittent advancing catheters were inserted to a depth of 6 mm. For all four groups, a 1 mm “decoring retraction” was used in an attempt to remove any agarose that may have become lodged in the catheters during insertion. The deployment distance, intrainfusion movement rates, and movement distances are summarized in Table 1. After the catheters were inserted, an indigo carmine dye solution (1% *w*/*w*) was infused at 1 µL/min for 100 min using a programmable syringe pump (Chemyx Fusion 100, Chemyx, Austin, TX, USA). The flowrate of 1 µL/min was chosen in order to minimize the potential of backflow and is the rage of flowrates range typically used in CED infusion studies [16,24,25,26,27,28,29,30,39,40,43]. Once the infusion was started, the continuously retracting or advancing catheters were moved 25 mm at a rate of 0.25 mm/min and the intermittent advancing catheters were inserted an additional 6 mm with 1 mm of retraction every 16 min, for a total additional insertion of 25 mm. The infusion line pressure was measured at 2 Hz using gage pressure sensors calibrated from 0 to 30 psig (0 to 1551 mmHg) and 0 to 1 psig (0 to 52 mmHg) (26PC series, Honeywell International Inc., Golden Valley, MN, USA). Pictures were taken at the start of the experiment and retaken every minute throughout the 100 min infusion.

**Figure 2:**
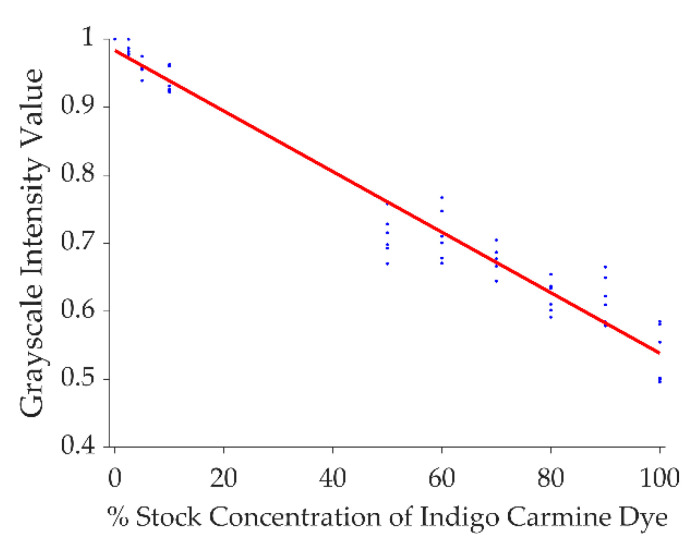
Grayscale intensity values plotted against 1% (*w*/*w*) indigo carmine dye dilutions in agarose gel used to determine the intensity threshold for converting grayscale images into binary images.

At the conclusion of the infusions, the images were post-processed using Matlab (Mathworks, Natick, MA, USA), as described previously [29,44]. First, the images were imported into Matlab and cropped to encapsulate only the surrounding area of infusion in the agarose gel. Images were then converted to binary using a threshold value of 0.8% of stock concentration. Concentration was determined based on a calibration conducted by diluting stock 1% (*w*/*w*) dye solution in deionized water and preparing agarose gel standards at 10 levels from 0% to 1.0% concentrations (*n* = 6/level). Pictures of the standards were taken and imported into Matlab. Each image was then cropped to a small area of the standard, then images were converted to grayscale and normalized to the 0% standard. The mean for each standard level was taken and a linear curve-fitting algorithm was used to create the model, which is shown in Figure 2.

Once each image was converted to binary, images were visualized and any artifacts that were present on the image were manually removed. Artifacts were defined as any geometry present on the binary image that did not contribute to infusion volume. The artifacts primarily consisted of small amounts of debris on the specimen container or presence of the catheter far outside of the infusion cloud. Next, the first image from each infusion was scanned to find the location of the catheter tip. Additionally, the angle of the catheter relative to the gel was determined. Then, *V*_d_ was calculated for two regions: the full dispersal region (*V*_d_) and forward dispersal (*V*_df_). For all groups except the continuous retraction group, *V*_df_ was defined as any infusion volume extending from the starting position of the catheter. For the continuous retraction group, *V*_df_ was defined as any infusion volume extending from the current location of the catheter tip toward the proximal end of the catheter at its present location. The location of the catheter tip was estimated based on the starting location of the catheter, the time elapsed, and the angle of catheter insertion. The radius, back flow distance, and forward flow distance were also calculated. The forward flow distance was defined as the axial length of *V*_df_ and the backflow distance was the axial length of the infusion that did not contribute to *V*_df_. Additionally, pressure and temperature readings were analyzed and experiments were discarded if either infusion line pressure eclipsed 50 mmHg or if the temperature strayed from a 25 ± 2 °C window. During the experiment, one of the temperature sensors began malfunctioning (affecting 3 infusions). However, since two separate infusions were conducted adjacent to one another at the same time and under the same conditions, the temperature from the adjacent infusions were used to evaluate exclusion criteria when temperature malfunctions occurred. A one-way analysis of variance was conducted to test for significant *V*_d_, *V*_df_, infusion radius, backflow distance, forward flow distance, and average pressure changes between groups. The analysis of variance was followed by a post-hoc Tukey’s test.

## 3. Results

A total of 35 experiments were conducted during this study. Seven stationary infusions were completed, with one outlier omitted from analysis because the pressure eclipsed 50 mmHg. Eight retraction infusions were performed, with two outliers omitted from analysis because the temperature strayed from the 2-degree window. Six intermittent insertion infusions were conducted, with none removed. Finally, 14 continuous insertion infusions were completed, with 13 omitted from analysis due to high infusion line pressure; due to lack of data (*n* = 1), the entire group was removed from further analysis.

**Figure 3:**
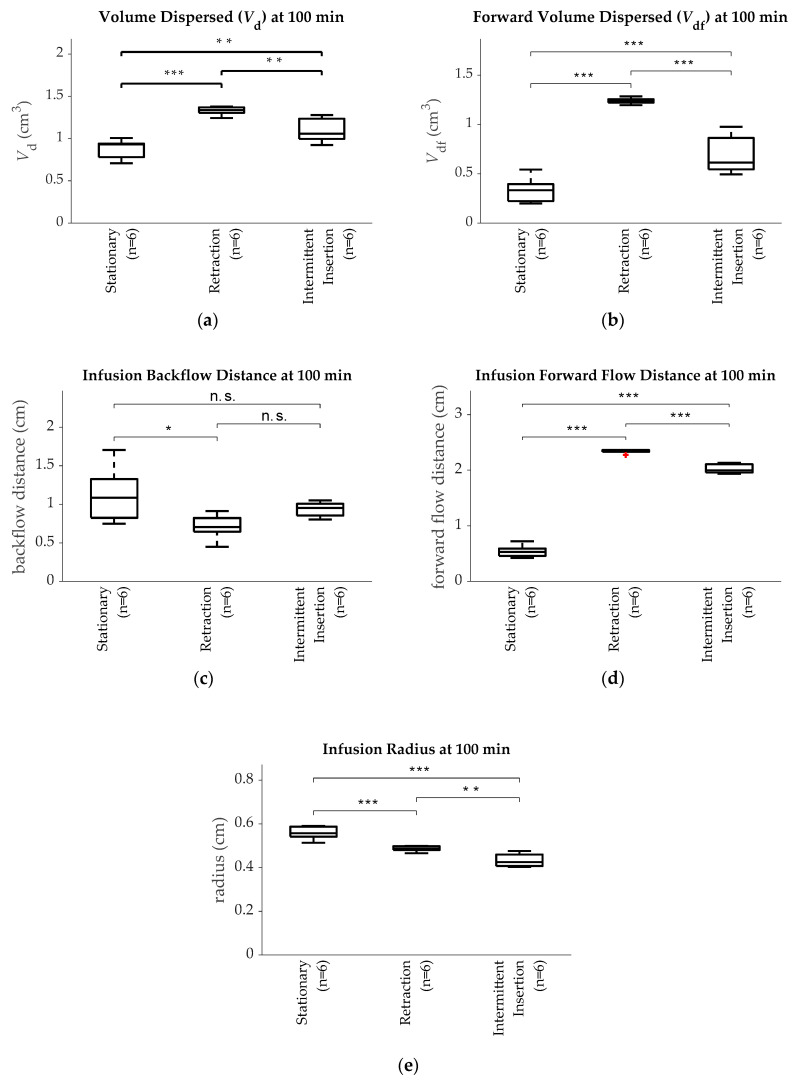
Plots of the (**a**) total volume dispersed (*V*_d_); (**b**) forward volume dispersed (*V*_df_); (**c**) backflow distance; (**d**) forward flow distance; and (**e**) infusion radius for the stationary, retraction, and intermittent insertion groups after 100 min of infusion. Note: * *p* ≤ 0.05; ** *p* ≤ 0.01; *** *p* ≤ 0.001

Volume Dispersed: A plot of *V*_d_ for each group after 100 min is shown in Figure 3a. The retracting group had the largest *V*_d_ (1.33 ± 0.05 cm^3^) of any group and was significantly larger than both the intermittent insertion group (1.09 ± 0.14 cm^3^; *p* < 0.01) and the stationary group (0.88 ± 0.11 cm^3^; *p* < 0.001). The intermittent insertion group had the second largest *V*_d_ and also had a significantly larger volume than the stationary group (*p* < 0.05).

Forward Volume Dispersed: The plot for *V*_df_ is shown in Figure 3b. Both the retracting (1.24 ± 0.03 cm^3^; *p* < 0.001) and intermittent insertion (0.68 ± 0.19 cm^3^; *p* = 0.001) groups had significantly more *V*_df_ than the stationary group (0.34 ± 0.13 cm^3^), and the retracting group had significantly more *V*_df_ (*p* < 0.001) than the intermittent insertion group.

Backflow and Forward Flow Distance: The backflow and forward flow distances for each group are plotted in Figure 3c,d respectively. The retracting group (0.71 ± 0.16 cm^3^) had less backward flow than the stationary group (1.13 ± 0.36 cm^3^; *p* < 0.05). No statistical significance was found between the intermittent insertion group (0.94 ± 0.09 cm^3^) and the other two groups (*p* > 0.2). Finally, both the retracting (2.33 ± 0.03 cm^3^) and the intermittent insertion (2.02 ± 0.08 cm^3^) groups had larger forward flow distances than the stationary group (0.54 ± 0.11 cm^3^; *p* < 0.001), with a larger forward flow distance in the retracting group than the intermittent insertion group (*p* < 0.001). In Figure 3d, the outlier was defined as 1.5 times the interquartile range under the first quartile. The outlier in the retraction group was calculated to be 1.6 times the interquartile range under the first quartile.

Infusion Radius: The infusion radius for each group after 100 min is shown in Figure 3e. The stationary group (0.56 ± 0.03 cm) had a small but significantly larger infusion radius than either the retracting (0.48 ± 0.13 cm; *p* < 0.001) or the intermittent insertion (0.43 ± 0.03 cm; *p* < 0.001) groups.

Infusion Line Pressure: The infusion line pressure of the retracting group (9.62 ± 3.20 mmHg) was lower than the stationary group (14.43 ± 4.71 mmHg), but not significantly so (*p* < 0.08). The intermittent insertion group (12.49 ± 3.07 mmHg) showed no significant differences in infusion line pressure from the other groups.

**Figure 4:**
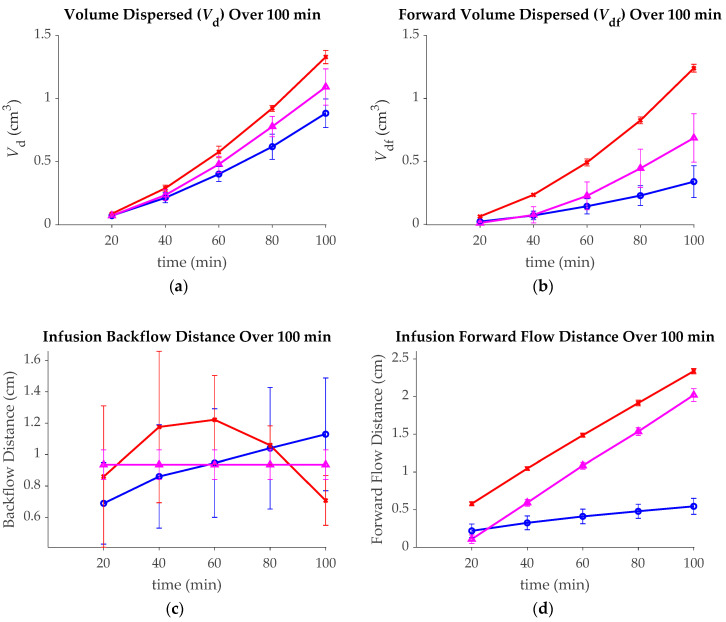

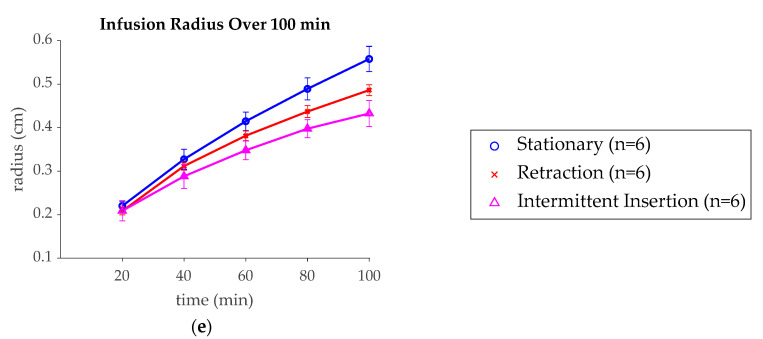
Plots of the (**a**) volume dispersed (*V*_d_); (**b**) forward volume dispersed (*V*_df_); (**c**) backflow distance; (**d**) forward flow distance; and (**e**) infusion radius for the stationary, retraction, and intermittent insertion groups at 20 min intervals throughout the 100 min infusion.

Data over infusion time: Figure 4 shows the outcome measurements at 20-min intervals through the 100-min infusion for each group. The retracting group had a higher *V*_d_ than either the stationary or intermittent insertion groups for all calculated time points, with significant levels (*p* < 0.01) of *V*_d_ at all time points after 20 min. The intermittent insertion group had a significantly higher *V*_d_ than the stationary group at the 80 and 100 min marks only (*p* < 0.01). Additionally, the retracting group had significantly greater forward volumes than either of the other groups at all time points (*p* < 0.001), and the intermittent insertion group had significantly greater *V*_df_ than the stationary group only at the 80- and 100-min time points (*p* <0.01). The stationary group had significantly higher infusion radii than the other groups at all time points greater than 60 min (*p* < 0.05), and the retraction group had significantly greater infusion radii than the intermittent insertion group at all time points after 20 min (*p* < 0.05). The stationary group only had significantly more backflow than the retracting group at the 100 min mark (*p* < 0.05). No significance was found for other groups or at other times. Lastly, the retracting catheter had significantly more forward flow distance than the other groups at all time points (*p* < 0.001). The stationary catheter had significantly more forward flow distance than the intermittent insertion group at the 20 min time point ( *p* < 0.05), but the reverse is true for all other time points during the infusion (*p* < 0.001).

**Figure 5:**
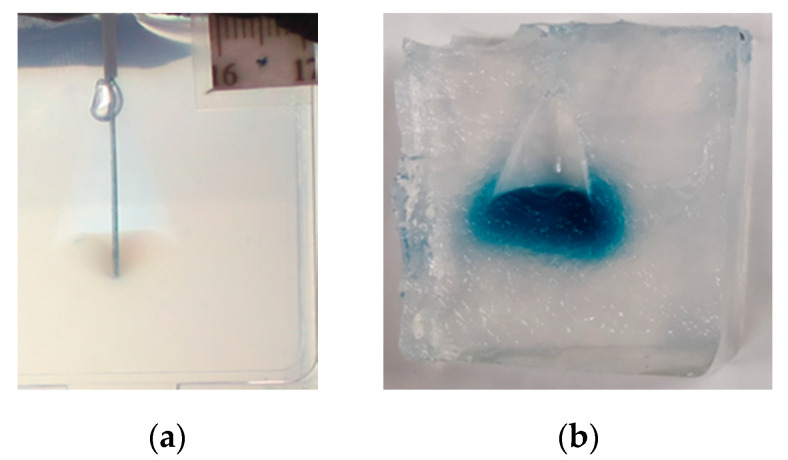
(**a**) Image of clogged continuously deploying catheter after 100 min of infusion. (**b**) Cross-sectional image of the same infusion after catheter removal.

Continuous Insertion: In all of the continuous insertion experiments where catheter clogging occurred (13 of 14), significant tearing of the gel occurred, leaving fluid-filled voids in the agarose in all areas except the location of the catheter tip, as shown in Figure 5.

## 4. Discussion

In this study, we examined the effect of stationary and moving catheters on the overall infusion volume, infusion radius, and backflow. We found a 1.5-fold increase in *V*_d_ with the retracting group and a 1.2-fold increase with the intermittently inserted catheter compared to the stationary control.

Both of the catheter movement groups had significantly larger total *V*_d_ values than the stationary group. The larger surface area over which infusion can occur created by catheter movement is likely the cause of the larger infusion volume; however, there is a tradeoff. The longitudinal infusion distance increases significantly with the moving groups, however the radius (horizontal infusion distance) decreases by a small but significant amount. Lewis et al. also found a significantly larger infusion volume and smaller infusion radii when using a recessed step catheter with large needle lengths placed before a reflux arresting step change, allowing for controlled reflux [38].

The reduction in radii found with both the retracting and intermittent insertion protocols could benefit from the use of catheters with multiple independent ports, such as the arborizing catheter that we previously developed [29]. A catheter with this design provides a reflux arresting geometry at the tip of the primary cannula and allows for multiple needles to be placed near each other. If the arborizing angle is created such that a small amount of overlap between individual needles occurs, thus preventing voids in the infusion, the increased volume associated with retracting catheters could be utilized.

The overall backflow distance for the retracting catheter was found to be significantly smaller than the backflow distance of the stationary catheter. This finding is in contrast to results found by Sillay et al. [35]. This could be due to the presence of the reflux-arresting feature used in this study. For both the intermittent insertion and retracting groups, the reflux-arresting feature was located approximately 5 mm from the catheter locations at the beginning and end of the infusions respectively. The close proximity of the reflux-arresting feature could be responsible for reducing the measured backflow distance. However, it should be noted that the backflow distance was statistically the same for all groups at earlier time points. Another possible cause for this apparent discrepancy is the method in which the backflow was calculated. Sillay et al. only reported backflow distances in groups where the forward flow distance was smaller than the backflow distance, while we reported all backflow distances. However, all of the stationary specimens had smaller forward flow distances than backflow distances, and for all groups the backflow distances were larger than the radii, with the exception of one retracting specimen.

In all specimens, dye never escaped the gel phantom. Further, dye was effectively arrested at or before the reflux arresting step change in all but two intermittent insertion specimens. The amount of reflux was also likely limited by the low flowrate chosen for the experiment, as reflux in the retracting catheter group did not reach the reflux arrestor until late in the experiment. The low flowrate and the quick insertion of the catheters at the beginning of the experiment likely limited agarose damage, as we expect dye would quickly reflux up the entire needle tract as soon as catheter movement began. While quick insertion rates limit damage to the agarose, and therefore reflux, Casanova et al. showed that slow insertion rates in rat brains result in less tissue damage and less backflow [45,46]. If both slow insertion speeds and slow retraction speeds are consistently used during continuous catheter retraction treatments, tissue damage may be mitigated and backflow can be reduced.

The infusion pressure for the retracting group was noticeably, but not significantly, lower than both the stationary and the intermittent insertion groups. High flowrate infusions have been shown to result in increased backflow [22,23]. However, the decreased pressure associated with the retracting catheters may allow for larger flowrates to be permissible, without the creation of backflow. An increase in the flowrate without overcoming a reflux-arresting step could have a considerable impact on the clinical relevance of large-volume CED infusions.

All but one of the continuous insertion experiments resulted in clogging of the catheter. Interestingly, other studies have started flow during the insertion process in order to limit clogging [27,35,36,47,48]. However, the insertion rate used in this experiment was much lower than that of other experiments, which may be partially responsible for these findings. Agarose buildup at the tip of the catheter was consistent with the slow insertion speeds in a study conducted by Casanova et al. [42]. Casanova et al. further found that the build-up of agarose (agarose damage) reduced the prestress on the tissue, allowing for more backflow. Given this finding and the presence of the large cavity in the agarose, it is likely that most of the infusion volume in this group would have been backflow, even if the catheter did not consistently clog.

*Study Limitations:* While agarose gel tissue phantoms have been used extensively to conduct CED experiments and have been shown to act as surrogates to the interstitial space in the brain tissue due to the gel’s poroelastic nature [49,50] and similar pore size to that of the brain [51], agarose gel tissue phantoms do not model either the cells or the blood vessels that infusions will typically follow [49]. Agarose gel tissue phantoms are homogenous and isotropic, which cannot capture the infusion morphology of pressure-driven flow inside of the heterogeneous and anisotropic brain. Additionally, the specific interactions of the catheter with the agarose gel during insertion or movement may not mirror that of the brain [48]. This hypothesis has been proven to a certain extent, as Casanova et al. demonstrated an inverse trend of tissue damage based on insertion speed in agarose gel compared to brain tissue [42,45]. However, it should be noted that studies utilizing multistep collinear payloads [32,37] did not note any adverse events due to intraoperative catheter movement. Nonetheless, additional work should be conducted that assess both the safety and efficacy of controlled catheter movement in vivo.

## 5. Conclusions

In this study, we assessed the effect of catheter movement on *V*_d_ in agarose gel phantoms. Catheters were inserted into phantoms and either kept stationary, continuously retracted, continuously deployed, or intermittently deployed during the infusion. Images of the infusion were taken and processed in order to determine the *V*_d_ of the infusion. Significantly higher *V*_d_ was found with both the retracting (1.5-fold) and the intermittent insertion (1.2-fold) groups compared to stationary catheters. The use of dynamic catheters could not only improve tumor coverage but could also allow for the creation of patient specific infusion geometries, furthering the efficacy of CED treatment.

## Figures and Tables

**Table 1:** Experimental group parameters.

Group	Initial Deployment Distance	Movement Direction	Movement Speed	Movement Distance	Number of Replicates
Stationary (Control)	30 mm	None	0 mm/min	0 mm	7
Continuously Retracting	30 mm	Backward	0.25 mm/min	25 mm	8
Continuous Insertion	6 mm	Forward	0.25 mm/min	25 mm	14
Intermittent Insertion	6 mm	Forward	600 mm/min	25 mm (5 mm every 16 min)	6
